# Preparation of Polystyrene/SiO_2_ Composite Aerogel Microspheres

**DOI:** 10.3390/ma19051036

**Published:** 2026-03-09

**Authors:** Zenghui Qian, Yangyang Yu, Wenjing Chen, Guodong Jiang, Yucai Shen, Zepeng Mao

**Affiliations:** College of Materials Science and Engineering, Nanjing Tech University, Nanjing 210037, China

**Keywords:** silica aerogel, microsphere, polymer crosslinking, hydrophobicity, structural enhancement

## Abstract

**Highlights:**

**What are the main findings?**

- Surface functionalization-coordinated enhancement via W/O emulsion & in situ copolym.
- The innovative application of a HMDS/KH570 binary hydrophobic modifier system.
- Developed Sty-DVB copolymerization for nanoscale reinforcement layer on aerogel microspheres.

**What are the implications of the main findings?**

- The study provides an efficient strategy for hydrophobic and structural enhancement of SiO_2_ aerogel.
- The application of dual hydrophobic modifiers demonstrates potential for tuning surface wettability.
- Nanoscale reinforcement layer enhances aerogel stability, expanding applications.

**Abstract:**

Silica aerogel microspheres demonstrate tremendous potential as fillers for diverse materials across various fields. Enhancing the strength of silica aerogel microspheres is therefore crucial for their practical applications. This study aims to develop novel hydrophobic polymer-reinforced silica aerogel microspheres using water glass as the precursor, hexamethyldisilazane (HMDS) as the modifier, and styrene as the crosslinking agent, with further strength enhancement achieved through short-term thermal post-treatment. The effects of varying polystyrene coating levels, crosslinker dosage, and short-term heat treatment on the structure and properties of silica aerogel were investigated. The optimized silica aerogel microspheres (Sample A-6) exhibited a specific surface area of 604.8 m^2^/g and a thermal conductivity of 0.030 W·m^−1^·K^−1^ and demonstrated excellent hydrophobicity and mechanical stability.

## 1. Introduction

Silica aerogel is a nanoporous material characterized by low density, ultralow thermal conductivity, high porosity, and high specific surface area. Within the aerogel, the bicontinuous 3D network formed by silica nanoparticles extends the heat conduction path and increases radiative heat loss [1,2,3,4]. Furthermore, nanopores with sizes smaller than the mean free path of air molecules effectively suppress gaseous convection [5,6,7]. Consequently, silica aerogels typically exhibit exceptional thermal insulation properties and are currently employed as thermal insulation and fireproofing materials in diverse fields including aerospace, petrochemicals, construction, and transportation [8,9,10,11,12]. However, their inherently low mechanical strength limits their broader application, while silica aerogels produced via the conventional sol–gel method are predominantly monolithic blocks [13,14,15,16,17]. If pulverized for use, this process may disrupt the internal aerogel structure, potentially compromising its thermal insulation performance [18,19,20,21].

Silica aerogel powders, such as microspheres or particles, offer advantages over monoliths by avoiding disruption of their internal structure inherent in processes such as mechanical fragmentation [22,23,24,25,26]. They also exhibit enhanced versatility in various applications. Their small size facilitates easier and faster modification and drying processes, effectively reducing manufacturing time [27,28,29,30]. In recent years, researchers have proposed several rapid synthesis techniques for silica aerogel powders. Pan et al. [31] synthesized a superhydrophobic (θ = 162°) aerogel powder using a water-glass-based sol–gel method at ambient pressure, incorporating mechanical fragmentation and filtration. Lee et al. [32] reported a technique for the rapid synthesis of silica aerogel powder using emulsion polymerization with water glass, n-hexane, and the surfactant Span 80.

To address the inherently low strength of aerogels, researchers have proposed modification strategies including the addition of organosilane precursors, fiber reinforcement, nanoparticle reinforcement, and organic polymer reinforcement. Yun et al. [33] prepared silica aerogels using methyltrimethoxysilane (MTMS) as the silicon source under ambient pressure drying. Testing showed that the Young’s modulus increased from 0.043 MPa to 1.102 MPa, with the aerogels exhibiting good thermal insulation (thermal conductivity of 0.036 W/(m·K)). However, the pores in these organic-modified aerogels are micrometer-scale, rather than strictly nanoscale. Li et al. [34] fabricated silica aerogel/aramid fiber composites using aramid fiber pulp as reinforcement, also via ambient pressure drying. The results indicated that compressive strength significantly improved with increasing aramid fiber content, reaching up to 1.2 MPa, while thermal conductivity ranged from 0.0232 to 0.0278 W/(m^−1^·K^−1^). Nevertheless, fiber reinforcement presents challenges. Firstly, short fibers tend to entangle and agglomerate, complicating the fabrication of uniform composites. Secondly, the relatively large fiber diameter compared to silica aerogel nanoparticles results in weak interfacial bonding and poor adhesion, making separation likely. Wu et al. [35] synthesized isocyanate-crosslinked silica aerogels using tetraethyl orthosilicate (TEOS) and (3-aminopropyl) triethoxysilane (APTES) as co-precursors and amine modifiers, with hexamethylene diisocyanate (HDI) as the polymer crosslinker. These aerogels achieved a thermal conductivity of 0.037 W/(m^−1^·K^−1^) and a remarkably high Young’s modulus of 18 MPa at 4.2% strain. The additional C-C covalent bonds between polymer molecules lead to wider interparticle necks and consequently higher specific stiffness and strength.

Therefore, in this work, water glass was used as the silicon source and employed the water-in-oil emulsion method to prepare silica aerogel microspheres. Then, we carried out surface hydrophobic modification on them. Subsequently, styrene, benzoyl peroxide, and divinylbenzene were copolymerized in ethanol solution. Because the copolymer is insoluble in ethanol, during the polymerization process, the copolymer will precipitate from ethanol and coat the surface of the microspheres as well as the internal three-dimensional network skeleton, thereby enhancing the structural strength of the silica aerogel microspheres.

## 2. Experimental Section

### 2.1. Materials

Sodium silicate (water glass, 33 wt%, energy band, 5.2–9 eV) was supplied by Yousuo Chemical Technology Co., Ltd., Jining, China. Hydrochloric acid (HCl, 99.5%), Span 80, Tween 80, and petroleum ether were purchased from Sinopharm Chemical Reagent Co., Ltd., Shanghai, China. Ammonium hydroxide (NH_3_·H_2_O, 98%), hexamethyldisilazane (HMDS), and styrene (99.9%) were purchased from Shanghai Aladdin Biochemical Technology Co., Ltd., Shanghai, China. Benzoyl peroxide (BPO, 99%) and divinylbenzene (DVB, 99.9%) were purchased from Shanghai Macklin Biochemical Co., Ltd., Shanghai, China. Absolute ethanol was purchased from Wuxi Yasheng Chemical Co., Ltd., Wuxi, China. Deionized water was supplied by Nanjing Wanqing Glass Co., Ltd., Nanjing, China.

### 2.2. Silica Aerogel Polymer Cross-Linked Coating Reinforcement

**Figure 1:**
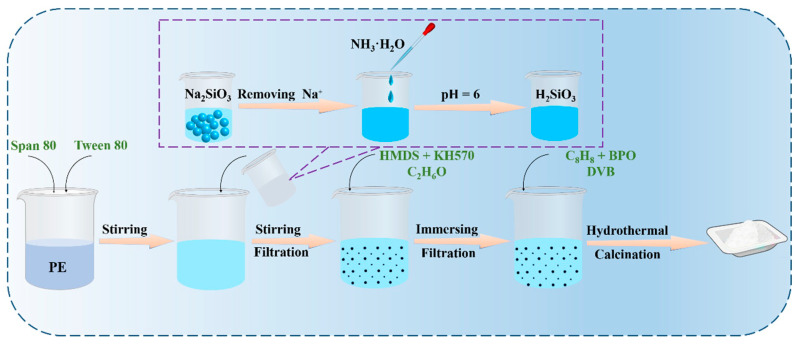
Preparation flowchart.

The overview of the preparation process is shown in Figure 1. Sodium silicate solution (4 wt% SiO_2_) was prepared by dissolving 0.37 mol (45.3 g) of water glass in 14.15 mol (254.7 g) of deionized water. Na^+^ ions were removed by passing the solution through a cation exchange resin column, yielding 230 g of silicic acid solution, to which ammonium hydroxide was then slowly added dropwise to adjust the pH to 6; 0.016 mol (7 g) Span 80 and 0.019 mol (0.8 g) Tween 80 were dissolved in 690 mL petroleum ether and stirred uniformly; the silicic acid solution was then added to the petroleum ether mixture and emulsified by high-speed stirring at 2000 rpm for 20 min (room temperature); gelation proceeded at 500 rpm for 6 h at 50 °C; after aging, the mixture was filtered to isolate SiO_2_ hydrogel particles; these hydrogel particles were immersed in 150 mL ethanol for solvent exchange with immersion cycles of twice for 1 h, twice for 3 h, and twice for 6 h; after filtration, SiO_2_ microgel particles (designated A-0) were obtained; 7.5 mL hexamethyldisilazane (HMDS) and 7.5 mL KH570 were mixed in 135 mL ethanol; A-0 particles were then immersed in this solution for 12 h for surface modification, yielding alkylated particles (A-1) after filtration; three separate ethanol solutions (40 mL each) containing styrene, benzoyl peroxide (BPO), and divinylbenzene (DVB) in a 10:1:2 ratio were prepared with monomer concentrations of 4.7 wt%, 8.8 wt%, and 12.5 wt%. Styrene, BPO and divinylbenzene were added to 40 mL of ethanol in a 50:5:1 ratio to make a mixed solution and stirred well. The surface alkylated SiO_2_ microsphere gel particles A-1 were added to the above three solutions for impregnation for 12 h and then reacted at 80–90 °C for 6 h in a hydrothermal reactor to obtain polymer-enhanced SiO_2_ microsphere gel particles A-2, A-3, A-4 and A-5 after filtration. The resulting gel particles were supercritical dried with carbon dioxide to obtain aerogel powder, and finally the aerogel particles numbered A-5 were placed in the muffle furnace for firing at temperatures of 210 °C and 230 °C, respectively, and the samples A-6 and A-7 were obtained by firing for 10 min.

### 2.3. Characterization

Thermogravimetric analysis (TGA) was performed on a Henven HCT-1 thermogravimetric analyzer (TGA550, TA Instruments, Shanghai, China) with a heating rate of 10 °C/min. Fourier transform infrared (FTIR) spectra were acquired using a Shimadzu IR-Spirit spectrometer (Shimadzu, Kyoto, Japan) equipped with an attenuated total reflection (ATR) accessory. The microstructure of the silica aerogel microspheres was characterized using field emission scanning electron microscopy (Nova NanoSEM 430, FEI, Brno-Královo Pole, Czech Republic/Eindhoven, The Netherlands). The thermal conductivity of silica aerogel microspheres was measured using a Hot Disk thermal constants analyzer (Hot Disk TPS 2500 S, Hot Disk Instruments, Gothenburg, Sweden). Specific surface area and pore size distribution (PSD) were estimated using a surface area and porosity analyzer (NOVA4200e, Quantachrome Instruments, Boynton Beach, FL, USA); samples underwent initial degassing at 90 °C for 10 h, and N_2_ adsorption–desorption isotherms were collected at 77 K. The specific surface area was calculated using the Brunauer–Emmett–Teller (BET) method, while pore size distribution was determined via the Barrett–Joyner–Halenda (BJH) method; total pore volume was estimated at p/p_0_ ≈ 0.99.

## 3. Results and Discussion

### 3.1. TG Analysis

**Figure 2:**
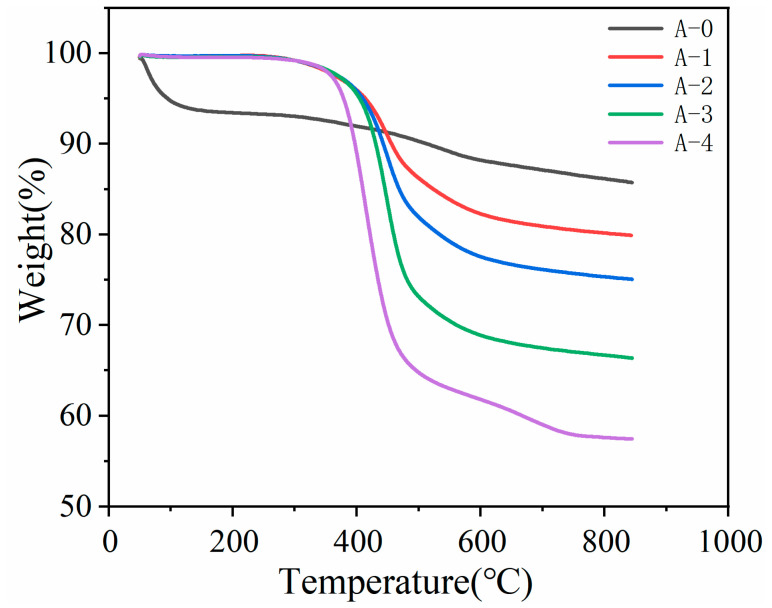
Thermogravimetric (TG) of silica aerogel microspheres.

Thermal stability is a key performance indicator for aerogel materials. This study systematically investigated the thermal decomposition behavior of SiO_2_ aerogel and its composite samples under nitrogen atmosphere using thermogravimetric analysis (TGA) (Figure 2). The TGA curves clearly reveal three characteristic weight loss stages, corresponding to the decomposition of distinct components within the materials. Initial weight loss stage (<200 °C): Desorption of adsorbed water. Unmodified sample A-0 exhibited significant mass loss in this region, primarily attributed to its surface containing numerous hydrophilic silanol (Si-OH) groups, which strongly adsorb water molecules from the environment via hydrogen bonding. In contrast, the hydrophobically modified samples (A-1, A-2, A-3, A-4) showed no obvious weight loss at this stage. This marked difference demonstrates the success of the hydrophobic modification: hexamethyldisilazane effectively reacted with surface Si-OH groups, replacing them with hydrophobic methyl (-CH_3_) groups, thereby drastically reducing the material’s surface energy and water adsorption capacity.

Major weight loss stage (200–600 °C): Decomposition of polystyrene (PS). The significant mass loss observed here originates primarily from the pyrolysis and volatilization of the PS component within the composite aerogels. PS decomposition typically begins with backbone scission, generating volatile products like styrene monomer and low-molecular-weight hydrocarbons. Notably, the cumulative weight loss percentage within this temperature range progressively increased from samples A-2 to A-4. This systematic change directly reflects the gradient increase in PS coating amount on the sample surfaces. This result not only quantifies the PS content differences among the samples but, more importantly, provides direct evidence for the successful coating of PS onto the SiO_2_ aerogel surfaces. The increase in weight loss rate perfectly aligns with the expected increase in coating amount.

Later slow weight loss stage (>600 °C): Decomposition of residual organic groups and structural densification. Beyond 600 °C, the curves plateaued, but slow mass loss continued. This stage’s weight loss mainly stems from the further oxidation or volatilization of residual minor organic components at high temperatures, such as short-chain alkyl groups from incomplete hexamethyldisilazane decomposition, trace hydroxyl groups, and potential carbonized residues. Ultimately, the samples reached a relatively stable mass, with the residual mass percentage reflecting the stability of the inorganic framework at high temperature. Typically, pure SiO_2_ aerogel exhibits a higher residual mass at these temperatures, whereas samples containing PS coatings show a correspondingly lower residual mass, associated with the complete volatilization of decomposed PS.

### 3.2. FTIR Analysis

**Figure 3:**
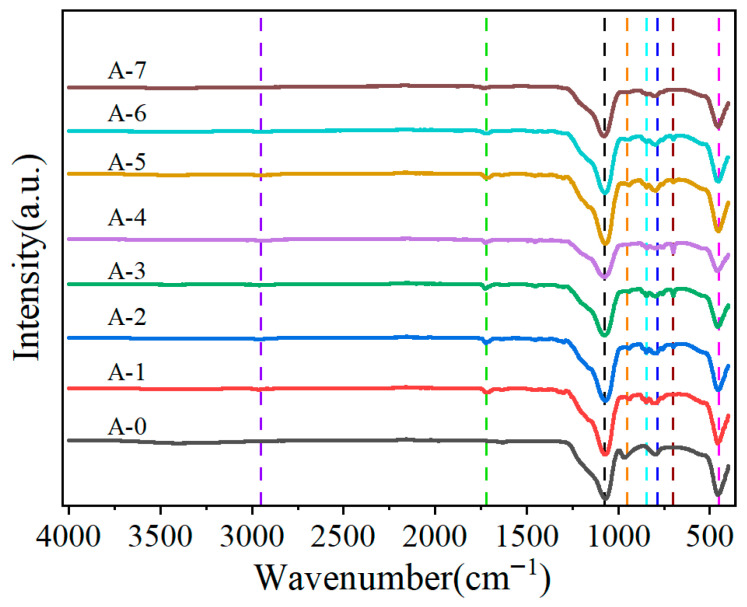
Infrared Spectroscopy (FTIR) of silica aerogel microspheres.

The chemical structure and surface functional groups of the samples were systematically characterized using Fourier transform infrared (FTIR) spectroscopy. As shown in Figure 3, all silica aerogel samples exhibited characteristic absorption bands near 450 cm^−1^, 787 cm^−1^, 951 cm^−1^, and 1077 cm^−1^, which are assigned to fundamental vibrational modes of the silica framework: the band at 450 cm^−1^ corresponds to the O-Si-O bending vibration; that at 787 cm^−1^ to the symmetric stretching vibration of Si-O bonds; that at 951 cm^−1^ to the in-plane stretching vibration of Si-OH groups (indicating surface hydroxyl presence); and the strong band at 1077 cm^−1^ originates from the asymmetric stretching vibration of Si-O-Si bridging bonds, collectively confirming the typical amorphous SiO_2_ network structure.

Critically, modified samples showed distinct additional peaks. A pronounced band near 2953 cm^−1^ is assigned to the C-H stretching vibration of -CH_3_ groups, providing direct spectroscopic evidence for successful replacement of surface silanols (Si-OH) by hydrophobic methyl (-CH_3_) groups during modification, indicating stable grafting. The characteristic peak observed at 1720 cm^−1^ stems from the C=O carbonyl stretching vibration, specifically indicating the introduction of the ester functional group from the silane coupling agent KH570 (γ-methacryloxypropyltrimethoxysilane). The absorption band near 845 cm^−1^ corresponds to the Si-C stretching vibration, further confirming covalent bonding (Si-O-Si or Si-C) between the silane coupling agent and the silica surface. The detection of a characteristic peak near 700 cm^−1^, attributed to the out-of-plane bending vibration of C-H bonds on the benzene ring, serves as a fingerprint region signature for the molecular structure of polystyrene (PS); its unambiguous appearance provides definitive spectroscopic evidence for the successful polymerization of styrene and its coating onto the silica aerogel microspheres.

In summary, FTIR analysis not only confirmed the structural features of the SiO_2_ matrix but also clearly revealed the chemical composition of the surface hydrophobic modification (methyl group introduction), coupling agent grafting (KH570), and polystyrene coating layer, establishing a solid structural characterization foundation for the material’s functional design.

### 3.3. NAD Analysis

**Figure 4:**
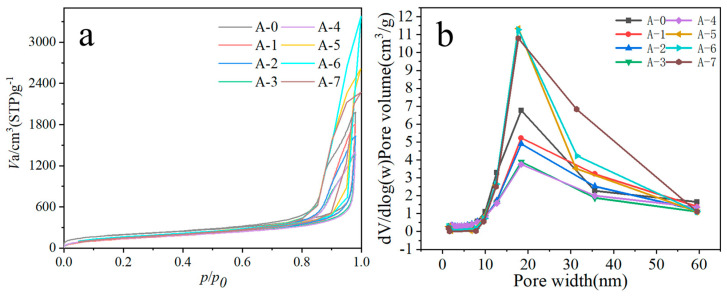
Adsorption/desorption isotherms and pore size distribution curves of RSAMs obtained from N_2_. (**a**) Adsorption/desorption isotherms; (**b**) pore size distribution.

The N_2_ adsorption–desorption isotherms of the RSAM series are shown in Figure 4a. All samples exhibit typical characteristics of mesoporous materials: low adsorption uptake in the low relative pressure region (p/p_0_ < 0.1), followed by a gradual increase with increasing relative pressure and a pronounced uptake at high relative pressures (p/p_0_ → 1.0). A clear adsorption–desorption hysteresis loop is observed for all samples. This behavior indicates multilayer adsorption followed by capillary condensation within mesopores and is consistent with the IUPAC classification of type IV isotherms. The presence of hysteresis further suggests that the pore structures are not ideally uniform or fully open but instead involve pore-neck restrictions and interconnected pore networks, which may correspond to ink-bottle or slit-shaped pore geometries.

The corresponding pore size distribution curves (Figure 4b) further confirm that the RSAMs are predominantly mesoporous. All samples display a pronounced main peak centered at approximately 20 nm, indicating that mesopores in this size range constitute the dominant pore structure. Noticeable differences in peak intensity and width are observed among the samples. In particular, samples A-6 and A-7 exhibit higher peak intensities together with a broadened tail extending toward the 30–40 nm range, implying a larger mesopore volume and a wider pore size distribution, possibly accompanied by the formation of larger mesopores or interparticle macropores. In addition, weak shoulders or minor peaks are detected in the low-pore-size region (<5 nm), suggesting the presence of a small fraction of narrow mesopores or micropores in some samples; however, their contribution is negligible compared with that of the dominant mesoporous framework.

The above pore characteristics can be rationalized in terms of the preparation process and subsequent treatments of the RSAMs. During the emulsion-templated gelation process, aqueous silicate droplets are dispersed in the oil phase under high-shear conditions, and these droplets are subsequently converted into SiO_2_ gel microspheres during gelation. The packing and interconnection of primary gel particles within the microspheres give rise to an interconnected pore network dominated by mesopores of intermediate size, accounting for the main pore size distribution peak centered at 20 nm. During surface modification, hydrophobic end-capping of surface silanol groups by HMDS effectively suppresses structural collapse induced by capillary stresses during solvent exchange and drying, while the organic coupling layer introduced by KH570 improves the interfacial compatibility between the inorganic silica framework and organic monomers, providing stable anchoring sites for the subsequent in situ polymerization.

After the introduction of styrene and crosslinking agents followed by thermally initiated polymerization, the formation mode of the polymer phase within and on the surface of the SiO_2_ gel microspheres plays a key role in the evolution of the pore structure. On the one hand, polymer bridging between primary particles enhances the mechanical stability of the gel skeleton, facilitating the preservation of the mesoporous network during supercritical CO_2_ drying. On the other hand, local enrichment or phase separation of the polymer phase may alter the geometry of interparticle pores, leading to a broadening of the pore size distribution toward larger mesopores. Furthermore, short-time low-temperature heat treatment promotes volumetric rearrangement and partial thermal evolution of the organic phase, accompanied by further condensation of the silica network. This combined effect enables secondary regulation of the mesoporous structure without destroying the overall spherical morphology, which is reflected by the increased contribution of larger mesopores in samples A-6 and A-7. The specific surface area and pore size data of each sample have been detailedly listed in Table 1.

Overall, the pore structure of the RSAMs originates from mesopores formed by the packing of primary particles induced by emulsion templating, while its tunability is governed by the synergistic effects of surface organic modification, in situ polymerization behavior, and subsequent thermal treatment. This multistep structural regulation mechanism provides a coherent explanation for the observed differences in N_2_ adsorption–desorption behavior and pore size distributions among the samples and offers a clear structural evolution pathway for the rational design of hierarchical mesoporous materials through process control.

### 3.4. MIP Analysis

**Figure 5:**
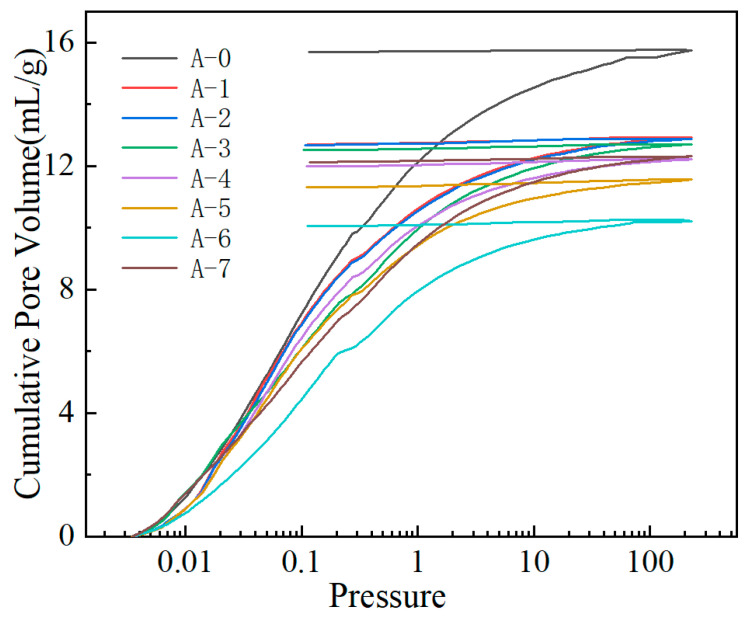
Mercury intrusion-extrusion curve of silica aerogel.

Mercury intrusion–extrusion porosimetry (MIP) was employed to elucidate the reinforcement effect induced by polymer modification on the pore structure and mechanical stability of the SiO_2_ microsphere aerogels. As shown in the intrusion–extrusion curves (Figure 5), all samples exhibit a monotonic increase in cumulative intrusion volume with increasing pressure; however, pronounced differences in curve shape, slope, and final intrusion volume are observed among the samples, reflecting systematic changes in pore accessibility and framework resistance to external pressure. Compared with the unmodified sample (A-0), the polymer-reinforced samples (A-2 to A-5) display a generally reduced cumulative mercury uptake and a more gradual intrusion slope over most of the pressure range. This behavior indicates that the in situ polymerization process partially occupies or narrows pore channels while simultaneously strengthening the silica skeleton through surface coating and interparticle bridging, thereby enhancing the resistance of the pore network against mercury penetration. The reinforcement mechanism can thus be interpreted as a synergistic effect of pore constriction and structural stiffening. On one hand, polymer deposition within the pore space decreases pore accessibility and effectively limits mercury intrusion. On the other hand, polymer bridges formed between primary silica particles improve load transfer and suppress local framework deformation under high pressure. These effects collectively manifest as a lower intrusion volume and a delayed mercury ingress, providing direct evidence of enhanced structural integrity induced by polymer modification.

Furthermore, the hysteresis between the intrusion and extrusion branches offers additional insight into pore geometry and elastic–plastic deformation behavior. In general, a large hysteresis loop is associated with ink-bottle–type pores or irreversible framework collapse during compression. The polymer-reinforced samples exhibit a modified intrusion–extrusion response, suggesting that polymer coating and bridging reduce the compressibility of pore necks and mitigate irreversible pore collapse under high pressure. This observation further supports the role of the polymer phase in stabilizing the pore framework and improving mechanical robustness. For the thermally treated samples (A-6 and A-7), changes in the MIP curves reflect the combined effects of polymer evolution and inorganic network rearrangement during short-term calcination. Thermal treatment may induce polymer densification, partial degradation, or shrinkage, leading to either pore tightening or the formation of localized voids and microdefects. Consequently, variations in cumulative intrusion behavior across different pressure regimes can be attributed to the balance between polymer removal and framework relaxation, which is consistent with the expected microstructural evolution derived from the preparation process.

Overall, the mercury intrusion–extrusion analysis provides compelling evidence that in situ polymer reinforcement effectively enhances the mechanical stability of SiO_2_ microsphere aerogels by reducing pore accessibility and strengthening the skeletal framework. The observed intrusion-extrusion behavior directly correlates with the polymer coating, interparticle bridging, and subsequent thermal evolution introduced during synthesis, thereby confirming the effectiveness of the designed reinforcement strategy.

### 3.5. FE-SEM Analysis

**Figure 6:**
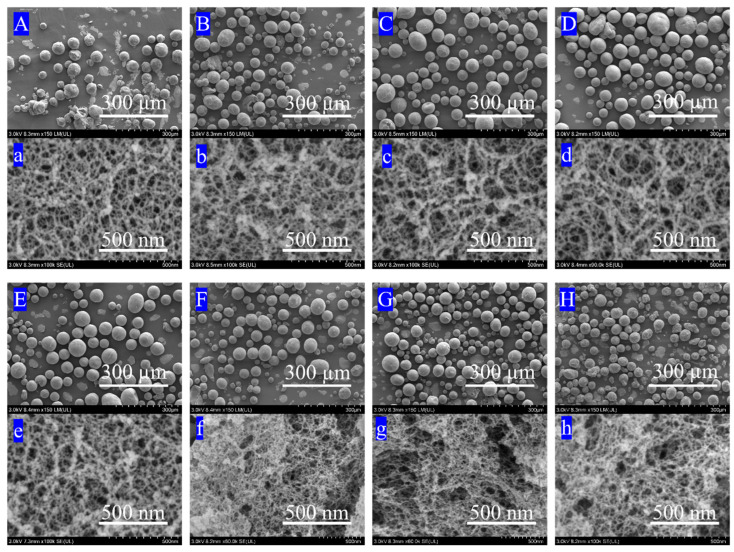
SEM of A-0 (**A**,**a**), A-1 (**B**,**b**), A-2 (**C**,**c**), A-3 (**D**,**d**), A-4 (**E**,**e**), A-5 (**F**,**f**), A-6 (**G**,**g**), A-7 (**H**,**h**).

The microscopic morphology and three-dimensional nanostructure of the samples were systematically resolved using field emission scanning electron microscopy (FE-SEM), and representative surface and fracture morphologies are shown in Figure 6. The surface morphologies (Figure 6A–E) clearly demonstrate the pronounced reinforcing effect of polystyrene (PS) crosslinking on the structural integrity of SiO_2_ microspheres. The uncoated sample A-0 (Figure 6A) displayed poor sphericity, a rough surface, and numerous fragmentation defects, indicating high mechanical fragility in pure SiO_2_ aerogel microspheres; surface-hydrophobically modified sample A-1 (Figure 6B) showed markedly reduced surface breakage and improved spherical regularity; samples A-2 to A-4, subjected to surface hydrophobic modification followed by PS coating, exhibited progressively smoother surfaces, significantly optimized sphericity, and virtually no structural damage with increasing PS coating amount (A-2 to A-4, Figure 6C–E), visually confirming that the PS coating effectively fills surface cracks and enhances microsphere resistance to fracture.

Fracture surface analysis (Figure 6a–e) further elucidated the PS crosslinking reinforcement mechanism at the nanoscale: comparing the fractures of A-0/A-1 (Figure 6a,b) with those of A-2/A-3/A-4 (Figure 6c–e) revealed that PS crosslinking substantially thickened the silica aerogel skeleton (e.g., increasing the diameter from the original d_0_ nm to d_4_ nm) and formed a denser three-dimensional network; this occurs because the PS polymer not only coats the skeleton surface but also bridges adjacent nanoparticles via molecular chains. As the crosslinker amount increased (A-2→A-4), the distribution breadth and connection density of the polymer at skeletal nodes simultaneously increased, thereby significantly enhancing mechanical interlocking and stress transfer efficiency between skeletal frameworks. This dual structural optimization, from “surface coating repair” to “skeletal crosslinking reinforcement”, ultimately endows the PS/SiO_2_ composite aerogels with exceptional mechanical stability, laying a robust structural foundation for subsequent functional applications.

Among the polymer-reinforced samples without thermal treatment, A-5 (Figure 6F,f) exhibits exceptionally high structural integrity and uniformity. The microspheres display nearly perfect sphericity with minimal surface fissures and very limited debris. High-magnification fracture images (Figure 6f) reveal a continuous and markedly thickened hybrid skeleton, in which primary SiO_2_ particles are extensively interconnected by polymeric ligaments accompanied by pronounced neck thickening, indicating effective reinforcement of the skeletal framework at this stage.

Notably, after short-time thermal treatment, sample A-6 (Figure 6G,g) achieves the most favorable balance between structural integrity, skeletal reinforcement, and pore-structure stability, and can therefore be identified as the sample with the optimal overall reinforcement effect in this series. Compared with A-5, A-6 retains excellent sphericity and a low degree of fragmentation, while its surface morphology evolves from a relatively dense polymer-covered state to a more homogeneous and stable hybrid interface, exhibiting only mild surface texturing without apparent structural damage. This behavior suggests that appropriate thermal treatment effectively induces volumetric rearrangement and interfacial densification of the polymer phase while avoiding excessive shrinkage or collapse of the silica skeleton.

Fracture surface analysis (Figure 6g) further confirms that A-6 possesses a continuous, uniform, and highly interlocked three-dimensional network composed of SiO_2_ skeletons and polymer phases. On the one hand, clear polymer bridging and skeletal thickening between primary particles are preserved, ensuring robust mechanical support; on the other hand, excessive accumulation of the organic phase is effectively mitigated, resulting in improved pore connectivity and a reduced presence of large or closed pores. This optimized architecture is particularly advantageous for simultaneously enhancing mechanical strength and maintaining a stable and accessible mesoporous network.

In contrast, further thermal treatment in A-7 (Figure 6H,h), although largely preserving the overall spherical morphology, leads to increased surface heterogeneity and the emergence of enlarged interparticle voids and localized cavities within the fracture structure. The reduced uniformity of the skeletal framework indicates that excessive thermal evolution begins to weaken the constraining effect of the polymer crosslinking network on the inorganic skeleton.

Taken together with the N_2_ adsorption–desorption and mercury intrusion porosimetry results, the SEM observations demonstrate that the reinforcement of RSAMs follows a stepwise structural evolution pathway of “surface coating, interparticle bridging/crosslinking, thermally induced structural reorganization”. Within this framework, A-6 represents the optimal compromise between skeletal strengthening, pore-structure stability, and mesoporous connectivity, providing a clear microstructural basis for its superior overall reinforcement performance.

### 3.6. Thermal Conductive Analysis

**Figure 7:**
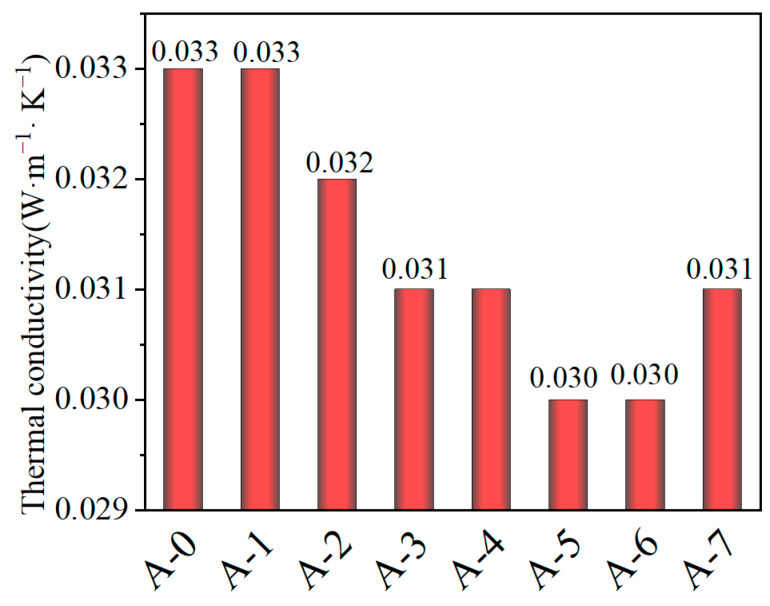
Thermal conductivity of silica aerogel.

As shown in Figure 7, the thermal conductivity of different samples exhibits notable variations, ranging from 0.030 to 0.033 W·m^−1^·K^−1^. The reference sample A-0 shows the highest thermal conductivity of approximately 0.033 W·m^−1^ K^−1^, indicating the presence of relatively continuous heat transfer pathways and limited microstructural regulation. Upon the introduction of modification or reinforcement strategies, the thermal conductivity of samples A-1 to A-4 gradually decreases. In particular, the values for A-2 and A-3 decrease to 0.032 and 0.031 W·m^−1^·K^−1^, respectively, suggesting that the incorporation of reinforcing components partially disrupts the original continuous thermal conduction network and increases interfacial thermal resistance.

Notably, samples A-5 and A-6 exhibit the lowest thermal conductivity, both approximately 0.030 W·m^−1^·K^−1^, with A-6 demonstrates the most pronounced overall reinforcement effect. This indicates that A-6 achieves the most effective microstructural regulation, likely due to a more uniform and refined pore structure or interfacial distribution, thereby efficiently suppresses heat transfer and significantly enhancing thermal insulation performance. In contrast, the thermal conductivity of A-7 slightly increases to 0.031 W·m^−1^·K^−1^, implying that excessive reinforcement or modification may introduce additional heat transfer pathways, thus compromising the insulation effect.

Overall, the thermal conductivity results are closely correlated with the reinforcement strategy and the resulting microstructural regulation of the materials. Among all samples, A-6 exhibits the best performance in reducing thermal conductivity, reflecting the optimal structural reinforcement and optimization, which provides a solid foundation for subsequent performance optimization and mechanistic analysis.

## 4. Conclusions

In this study, silica aerogel microspheres derived from sodium silicate were successfully reinforced through a combined strategy of functionalization and in situ polymerization. Dual modification with HMDS and KH570 effectively reduced the density of surface hydroxyl groups and enhanced interfacial compatibility, facilitating the uniform deposition of a polystyrene/divinylbenzene (PS/DVB) network through solvent-induced phase separation. Structural and thermal analyses confirmed that the polymer coating improved the mechanical integrity of the aerogel framework while preserving its mesoporous structure. Under optimized synthesis conditions, the resulting silica aerogel microspheres (Sample A-6) exhibited a high specific surface area of 604.8 m^2^·g^−1^, a low thermal conductivity of 0.030 W·m^−1^·K^−1^, along with excellent hydrophobicity and mechanical stability. Although polymer incorporation led to a moderate reduction in surface area and pore volume, an appropriate balance between structural reinforcement and thermal insulation performance was achieved. In contrast excessive thermal treatment induced structural heterogeneity. Overall, this synergistic modification strategy offers an effective route to enhance both the robustness and insulation performance of silica aerogel microspheres, highlighting their promising potential for practical thermal insulation and composite material applications.

## Figures and Tables

**Table 1:** Characterization Data of Silica Aerogel Microspheres.

	A-0	A-1	A-2	A-3	A-4	A-5	A-6	A-7
Specific surface (m^2^/g)	674.0	581.1	566.3	542.8	538.8	589.1	604.8	560.8
Pore volume (cm^3^/g)	3.065	2.783	2.529	2.116	2.163	4.037	5.226	3.509
Particle size (nm)	45.63	44,8	42.1	43.16	46.9	45.1	43.6	45.3
Thermal conductivity (W·m^−1^ K^−1^)	0.033	0.033	0.032	0.031	0.031	0.030	0.030	0.031

## Data Availability

The raw data supporting the conclusions of this article will be made available by the authors on request.

## References

[1] Baimova J.A., Shcherbinin S.A. (2023). Strength and Deformation Behavior of Graphene Aerogel of Different Morphologies. Materials.

[2] Chen H., Deng Q., Hu B., Gao Y. (2022). Flexible curdlan-based aerogels enhanced by wood fibers with ultralow thermal conductivity. Thermochim. Acta.

[3] Chen Q., Wang H., Sun L. (2017). Preparation and Characterization of Silica Aerogel Microspheres. Materials.

[4] Dayarian S., Yang L., Majedi Far H. (2023). Development of polyimide aerogel stock shapes through polyimide aerogel particles. J. Porous Mater..

[5] Effraimopoulou E., Jaxel J., Budtova T., Rigacci A. (2024). Hydrophobic Modification of Pectin Aerogels via Chemical Vapor Deposition. Polymers.

[6] Feng J., Zhuang Z., Zhou Y., Li C. (2024). Highly Conductive PEDOT:PSS Aerogels. Adv. Funct. Mater..

[7] Ganonyan N., He J., Temkin A., Felner I., Gvishi R., Avnir D. (2021). Ultralight monolithic magnetite aerogel. Appl. Mater. Today.

[8] Ge L., Shang S., Ma Y., Koudama T.D., Yuan K., Liu W., Cui S. (2025). Overview of Aerogels for Thermal Insulation. ACS Appl. Mater. Interfaces.

[9] Hosseini M., Rahmanian V., Pirzada T., Frick N., Krissanaprasit A., Khan S.A., LaBean T.H. (2022). DNA aerogels and DNA-wrapped CNT aerogels for neuromorphic applications. Mater. Today Bio.

[10] Kantor Z., Wu T., Zeng Z., Gaan S., Lehner S., Jovic M., Bonnin A., Pan Z., Mazrouei-Sebdani Z., Opris D.M. (2022). Heterogeneous silica-polyimide aerogel-in-aerogel nanocomposites. Chem. Eng. J..

[11] Laskowski J., Milow B., Ratke L. (2016). Aerogel–aerogel composites for normal temperature range thermal insulations. J. Non-Cryst. Solids.

[12] Li C., Zhang D., Ren W. (2021). Phase Change Materials Composite Based on Hybrid Aerogel with Anisotropic Microstructure. Materials.

[13] Li H., Chen Y., Liu L., Hu X., Sun H., Li Y., Wu X., Wang J. (2025). A general strategy for dramatic enhancement of aerogels via an aerogel-functionalization-aerogel approach. J. Mater. Chem. A.

[14] Li X., Zhang X., Zhang H., Sun X., Mu Y., Barrett T., Doyle C., Minus M.L., Zheng Y. (2024). Transparent and Flexible Hierarchical Porous Structure of Polyvinyl Alcohol Aerogel: A Microstructure Study. Materials.

[15] Liu S., Wang S., Shuai S., Weng Y., Zheng F. (2023). Efficient Solar Desalination of Seawater Using a Novel Carbon Nanotube-Based Composite Aerogel. Materials.

[16] Luo Y., Li L., Su Z., Yan A., Tian H., Cao Y., Niu B., Long D. (2025). High-strength and superior thermal insulating SiOC ceramic aerogels derived from thermostable silicone aerogels. Ceram. Int..

[17] Paraskevopoulou P., Chriti D., Raptopoulos G., Anyfantis G.C. (2019). Synthetic Polymer Aerogels in Particulate Form. Materials.

[18] Sonu S.S., Rai N., Chauhan I. (2023). Multifunctional Aerogels: A comprehensive review on types, synthesis and applications of aerogels. J. Sol-Gel Sci. Technol..

[19] Smirnova I., Gurikov P. (2017). Aerogels in Chemical Engineering: Strategies Toward Tailor-Made Aerogels. Annu. Rev. Chem. Biomol. Eng..

[20] Sozcu S., Venkataraman M., Wiener J., Tomkova B., Militky J., Mahmood A. (2023). Incorporation of Cellulose-Based Aerogels into Textile Structures. Materials.

[21] Su L., Jia S., Ren J., Lu X., Guo S.-W., Guo P., Cai Z., Lu D., Niu M., Zhuang L. (2023). Strong yet flexible ceramic aerogel. Nat. Commun..

[22] Sun W., Yu L., Su J., Liu R., Yan X., Su D., Zhang P., Li X. (2024). Preparation of SiO_2_ aerogel by water glass: Effect of different sodium removal methods on aerogel properties. J. Sol-Gel Sci. Technol..

[23] Tong Z., Yan B., Zhang B., Xu H., Li X., Ji H. (2021). Preparation and textural evolution: From organosilane aerogel to SiOC aerogels. Ceram. Int..

[24] Wang C., Eisenreich F., Tomović Ž. (2024). Aerogel-To-Sol-To-Aerogel (ASA) Process for Recycling, Repairing, Reprogramming of High-Performance Organic Aerogels. Adv. Funct. Mater..

[25] Wang J., Wang J., Sheng Z., Du R., Yan L., Zhang X. (2021). Solid–Liquid–Vapor Triphase Gel. Langmuir.

[26] Wang T., Zhang H., Tang S., Jia C. (2024). Multifunctional MXene composite aerogels modified via hyperbranched gels. J. Mater. Chem. A.

[27] Wang W., Pang L., Jiang M., Zhu Y., Wang F., Sun J., Qi H. (2022). Fabrication of SiCN(O) Aerogel Composites with Low Thermal Conductivity by Wrapping Mesoporous Aerogel Structures over Mullite Fibers. Materials.

[28] Wang W., Tong Z., Li R., Su D., Ji H. (2021). Polysiloxane Bonded Silica Aerogel with Enhanced Thermal Insulation and Strength. Materials.

[29] Yu Z.L., Qin B., Ma Z.Y., Huang J., Li S.C., Zhao H.Y., Li H., Zhu Y.B., Wu H.A., Yu S.H. (2019). Hard Carbon Aerogels: Superelastic Hard Carbon Nanofiber Aerogels (Adv. Mater. 23/2019). Adv. Mater..

[30] Zou F., Budtova T. (2023). Starch Alcogels, Aerogels, and Aerogel-like Xerogels: Adsorption and Release of Theophylline. ACS Sustain. Chem. Eng..

[31] Pan Y., He S., Cheng X., Li Z., Li C., Huang Y., Gong L. (2017). A fast synthesis of silica aerogel powders-based on water glass via ambient drying. J. Sol-Gel Sci. Technol..

[32] Lee K.J., Kim Y.H., Lee J.K., Hwang H.J. (2018). Fast Synthesis of Spherical Silica Aerogel Powders by Emulsion Polymerization from Water Glass. ChemistrySelect.

[33] Yun S., Guo T., Zhang J., He L., Li Y., Li H., Zhu X., Gao Y. (2017). Facile synthesis of large-sized monolithic methyltrimethoxysilane-based silica aerogel via ambient pressure drying. J. Sol-Gel Sci. Technol..

[34] Li Z., Gong L., Cheng X., He S., Li C., Zhang H. (2016). Flexible silica aerogel composites strengthened with aramid fibers and their thermal behavior. Mater. Des..

[35] Wu X., Man J., Liu S., Huang S., Lu J., Tai J., Zhong Y., Shen X., Cui S., Chen X. (2021). Isocyanate-crosslinked silica aerogel monolith with low thermal conductivity and much enhanced mechanical properties: Fabrication and analysis of forming mechanisms. Ceram. Int..

